# Knowledge and interactions of the local community with the herpetofauna in the forest reserve of Quininí (Tibacuy-Cundinamarca, Colombia)

**DOI:** 10.1186/s13002-020-00370-8

**Published:** 2020-04-15

**Authors:** Juan Camilo Ríos-Orjuela, Nelson Falcón-Espitia, Alejandra Arias-Escobar, María José Espejo-Uribe, Carol Tatiana Chamorro-Vargas

**Affiliations:** 1 Museu de Zoologia da Universidade de São Paulo, Avenida Nazaré 481, Ipiranga, São Paulo, SP CEP 04263-000 Brazil; 2 Grupo de Morfología y Ecología Evolutiva, Instituto de Ciencias Naturales, Universidad Nacional de Colombia, Sede Bogotá, Apartado, 7495 Bogotá D.C., Colombia; 3 Laboratorio de Ecología Evolutiva, Departamento de Biología, Facultad de Ciencias, Universidad Nacional de Colombia, Sede Bogotá, Ciudad Universitaria, Bogotá D.C., 11001 Colombia; 4 Grupo estudiantil de Herpetología, Área curricular de Biología, Facultad de Ciencias, Universidad Nacional de Colombia, Sede Bogotá, Ciudad Universitaria, Bogotá D.C., 11001 Colombia; 5 Grupo de Biodiversidad y Conservación Genética, Instituto de Genética, Universidad Nacional de Colombia, Sede Bogotá, Ciudad Universitaria, Bogotá D.C., 11001 Colombia

**Keywords:** Ancestral knowledge, Ethnoherpetology, Herpetofauna, Interactions, Local communities

## Abstract

**Background:**

The study of human-nature relationship has made possible to understand the life dynamics of the communities and the biodiversity with which they cohabit. Although there has been a rise of ethnobiological studies over the last decade, little is known about human interaction with herpetofauna in South America and Colombia. In this work, we analyzed the knowledge, perception, and interaction of a local community located in the forest reserve of Quininí (RFPCQ) in Cundinamarca (Colombia), concerning to the herpetofauna that inhabits the area.

**Methods:**

We performed semi-structured surveys containing 30 questions categorized into three groups: academic knowledge (1), use and cultural beliefs (2), and interactions (3) related to the herpetofauna that occurs in the region. The obtained data in question groups 1 and 2 are presented as a qualitative summary. For the question group 3, we assigned the answers to a hostility value according to the possible reaction of each individual interviewed in a hypothetical encounter with the herpetofauna and built tendency charts in order to see the positive or negative reactions due to the birthplace (urban/rural) and gender (male/female).

**Results:**

The community recognized the presence of amphibians and reptiles that cohabit their space, as well as their potential habitats. Besides, the role of herpetofauna was recognized in the magical/religious traditions for some inhabitants of the region, mainly associated with the fate and cure of chronic diseases. In general, the perception of amphibians and reptiles varied according to the origin and gender of the people, which tend to have a more positive perception of reptiles than compared to amphibians in most cases.

**Conclusions:**

Although there was a general lack of knowledge on the part of the inhabitants of the RFPCQ about the biological and ecological aspects of herpetofauna, the population recognized the basic information about the habitats of these animals within the reserve area. There is a wide variety of uses of amphibians and reptiles in traditional medicine. Greater efforts should be made in the transmission and dissemination of knowledge about the ecological functions of herpetofauna.

## Background

Human communities have established a close relationship with the herpetofauna with which they cohabit, based on the use and understanding of amphibians and reptiles [1–5]. Thereby, the ethnoherpetology (as an integral part of ethnobiology) studies traditional knowledge acquired from relationships between human-herpetofauna and interactions themselves, such as how a social group classifies and identifies different amphibians and reptiles species and their traditional uses [6].

Colombia is one of the most diverse countries of the world regarding herpetofauna species; currently, 616 species of reptiles and 835 species of amphibians [7, 8] have been identified in Colombia, with 115 and 367 endemic species, respectively [9]. Considering this diversity, it is important to understand local communities’ perceptions about the species with whom they share their territory to reveal information about the historic relationship of these communities with nature in the region. This can be the base for establishing conservation plans at local and regional scale, involving academics, politics, and local communities [10].

For some years now, ethnozoological studies have been conducted in Colombia that focused mostly on reptiles with high commercial usage, e.g., *Trachemys callirostris* (Gray, 1856), *Iguana iguana* (Linnaeus, 1758), *Boa constrictor* Linnaeus, 1758 and *Tupinambis teguixin* (Linnaeus, 1758), among others [11–14]. Nevertheless, there is little information about the use and perception of herpetofauna as a biological group, which is concerning when the high biological diversity of Colombia and Andes mountains is taken into account [9, 15, 16]. Thus, efforts to obtain information about the fauna and its interactions within the habitat become relevant where ethnobiological studies play a fundamental role in understanding the recent ecosystem dynamics, such as changes in the vegetal covertures and biodiversity among others [17, 18]. The *Reserva Forestal Protectora Cerro Quininí* (RFPCQ) forest reserve is a conservation area located in the Sumapaz province (Cundinamarca, Colombia), which is one of the most important biological corridors not only for the region but also for the eastern Andes, known as the Chingaza-Sumapaz-Guerrero corridor [19]. Besides, records of archeological remains, such as petroglyphs and passage routes, associated with the Panche community, an indigenous culture that inhabited this territory in pre-colonial times, are known [20, 21].

Due to its cultural and biological importance, RFPCQ has been the focus of the studies in diversity [22–27], ecology [28–30], anthropology [20, 21, 31–33], rural development, and innovation in production systems [34, 35], among others. Although most of the research associated with RFPCQ remains unpublished literature, it has actively involved community participation, which allowed them to appropriate environmental aspects. These initial approaches to environmental problems constitute a fundamental basis for understanding the current dynamics of the ecosystems to design a conservation plan where the community has a direct role.

Taking this into account, our aim is to evaluate the knowledge and perception of the RFPCQ community about the herpetofauna in three different aspects: (1) academic knowledge, (2) use and cultural beliefs, and (3) human-herpetofauna interactions. In this way, we propose an initial approach to the relationship between the community and amphibians and reptile species that inhabit their territory.

## Methods

### Study area

**Fig. 1:**
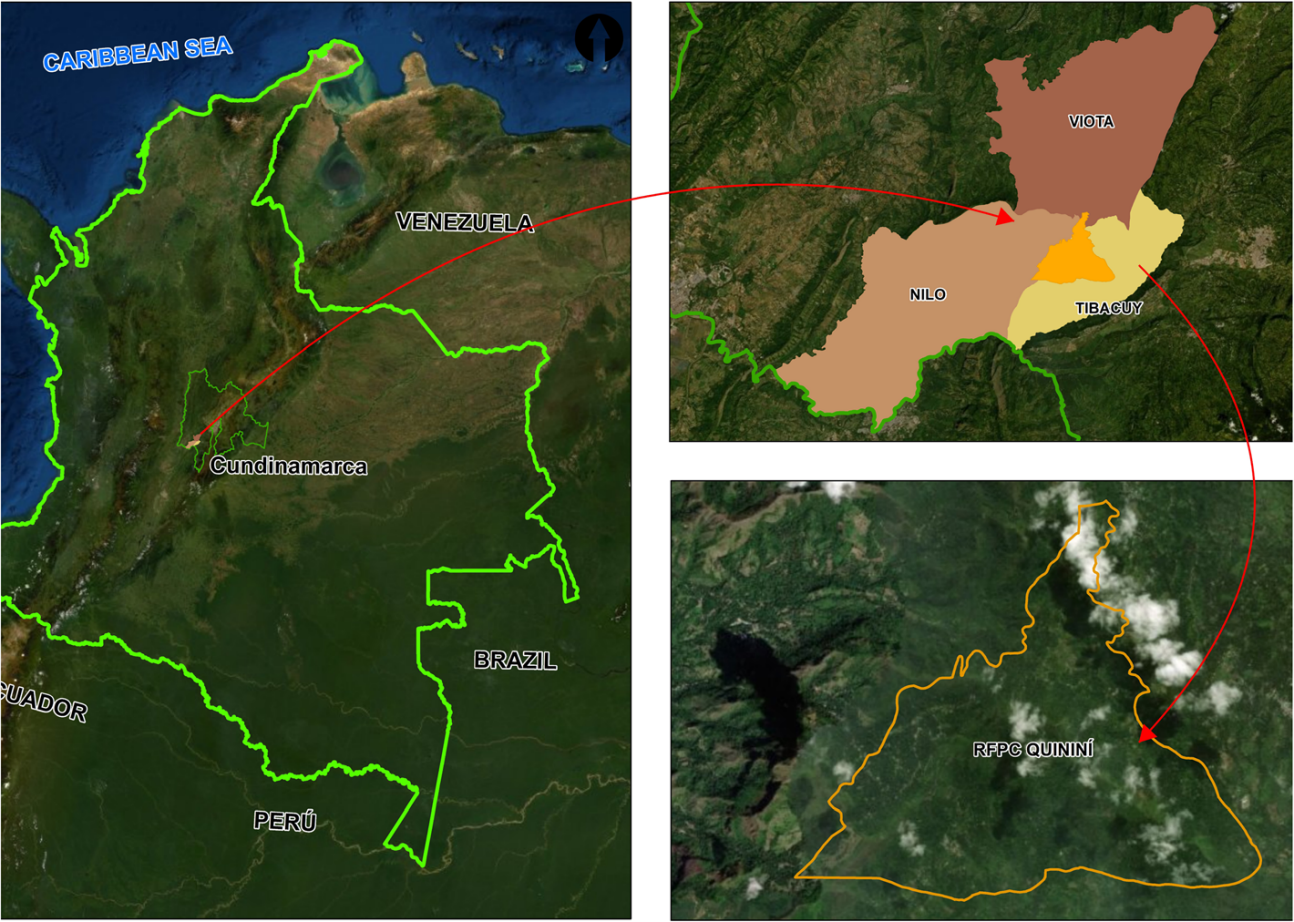
Localization of *Reserva Forestal Protectora Cerro Quininí* (RFPCQ) forest reserve

The *Reserva Forestal Protectora Cerro Quininí* (RFPCQ) forest reserve is located in Tibacuy, Nilo and Viotá municipalities (Cundinamarca, Colombia) (Fig. 1), between 1050 and 2133 m above sea level on Eastern Andes range. The region has a predominantly temperate climate, with an average annual temperature of 19.2 °C and a bimodal rainfall pattern [18]. With an approximate area of 1947 ha, it is one of the most extensive conservation areas in the Cundinamarca department [19].

According to the classification of Holdridge (1947) [36], the RFPCQ is located in the life zones of lower mountain moist forest and, to a lesser extent, lower mountain dry forest. Despite its protected area status, about 90% of the land associated with the RFPCQ are private properties, mainly with mixed crops, grazing areas, and coffee crops, being the largest [18, 35, 37, 38].

The area was declared as a protected forest reserve in 1987 to conserve its renewable natural resources and environment by request of the people inhabiting the area, mainly because of essential water springs, which supply the region [39].

### Data collection

Following Margoluis and Salafsky (1998) and Drumond et al. (2009) [40, 41], we made 61 semi-structured surveys during April 2016 and June 2019 with randomly selected respondents in the communities of Cumaca inspection, Albania, and La Vuelta villages (Tibacuy municipality), gathering information from people in urban as well as rural areas near the RFPCQ. We were unable to extend our surveys to more people due to the large distance between homes in the rural area and its low density of inhabitants.

Each survey had 30 questions of which 12 were related to general topics of amphibians and 18 were about general topic reptiles. We collected demographic information such as name, gender, age, occupation, birthplace, and education level in the following categories: illiterate, fundamental basic education, secondary education, and university education. Following the International Society of Ethnobiology Code of Ethics (2006) [42], before we started each survey, the project aims were presented and the consent to use the information was requested from each person. The complete model of the survey is available as an additional file (see Additional file 1).

Questions were categorized into three groups: academic knowledge (1), use and cultural beliefs (2), and interactions (3). The first group refers to questions related to the biology and ecology of the herpetofauna; in the second group, the questions were related to the ancestral knowledge of the community about the local herpetofauna and the third group contains questions about perception and human-herpetofauna interactions in daily activities.

### Data analysis

The obtained data in question groups 1 and 2 are presented as a qualitative summary. For the data concerning question group 3, answers were assigned to a hostility value according to the possible reaction of the individual interviewed in a hypothetical encounter with herpetofauna. In this way, interaction with amphibians was classified as a negative (hostile) behavior when respondents suggested trying or scare the animal; neutral behaviors when observing the animal and staying still or moving away; and positive behaviors when transporting the animal to a nearby body of water. Interaction with reptiles was classified in the same way, except for the behavior to remain still or move away, which was considered positive.

Demographic information obtained from the surveys was organized in categories for each variable. Age was categorized into three groups: (a) young people (14-26 years old), (b) adults (27-51 years old), and elder (52-83 years old). Besides, the data obtained in the urban area of Cumaca were considered as “urban,” whereas the data obtained in the villages of Albania and La Vuelta were considered as “rural.”

We also built trend-charts using data from question group 3 to see the positive or negative reactions due to the birthplace (urban/rural) and gender (male/female). We selected birthplace and gender because the initial analysis showed that these two factors had the biggest expected difference in the population.

To compare the samples, a qualitative analysis between the groups was used to assess differences in the answers given the birthplace, education level, and gender; this was done for questions from group 1 (academic knowledge) and 2 (cultural uses and beliefs). The localization map of the study area was built with ArcGIS 10.5 [43].

## Results

We surveyed 61 respondents, aged 14-83 with a balanced gender ratio (1:1). Table 1 shows the distribution of the population surveyed in the education level completed and occupation categories.

**Table 1:** Distribution of the population surveyed into completed education and occupation categories

	Female	Male	Total
**Education level**			
High school	16	16	32
Primary school	8	10	18
College degree	4	2	6
Graduate degree	1	1	2
Technical studies	1	0	1
Illiterate	0	1	1
Unanswered	0	1	1
**Occupation**			
Employee, merchant or independent	8	8	16
Student	7	9	16
Agriculture and land management work	4	10	14
Housework and other trades	11	3	14
Unemployed	0	1	1

### Academic knowledge

Regarding amphibians, 33 people (more than half of the respondents) classified frogs and toads within this group; six people classified them as reptiles, whereas 22 people said they did not know. Also, they recognized water reserves as a habitat for amphibians, as well as its importance in their life and reproduction cycles. However, only eight people associated amphibian reproduction with soft egg posture and the presence of larval stages. Most respondents described wet soil, plants with water reserves, and crops as a preferred habitat for frogs and toads. Regarding their ecological function, respondents recognized the importance of amphibians as pest controllers and associated their presence with the reduction of mosquitoes’ abundance. Although most of the respondents (43 people) recognized the existence of poisonous frogs, only two persons stated that it is possible to find them in Colombia.

As for reptiles, 36 people classified lizards and snakes within this group, three persons classified them as amphibians, whereas 22 people said they did not know. According to the surveyed population, the main habitats of reptiles are associated with tall trees, crops, grasslands, and wet soils. Although the role reptiles play within the ecosystem is unclear, nine people knew that they can act as pest controllers.

Fifty-three of the respondents acknowledged that there are venomous snakes in the region. However, only 21 people of the population surveyed said they knew the medical treatment associated with snake bites, such as the use of antiofidic serum and the referral to the municipal medical services.

### Use and cultural beliefs

For 39 respondents, amphibians are not part of their cultural or magical-religious beliefs, while three of them said they did not know. On the other hand, 19 people of the surveyed population described cultural beliefs and uses in traditional medicine associated with this group (Table 2). Twenty-two people of the surveyed population perceive amphibians as dangerous animals, whereas 13 people describe them as causes of skin diseases such as sores, rash, allergies, inflammations, and even necrosis.

**Table 2:** Uses and cultural beliefs of amphibians and reptiles identified in the RFPCQ region

Group	Species	Use or cultural belief
Amphibians	Frogs and toads (general)	“The intestines are used to make threads for wounds suture.”
		“By rubbing the frogs, you can cure the varicose vein.”
		“Toads cause sores, rash, and allergies. Toad’s urine causes inflammation and skin necrosis.”
		“Some people look for good luck numbers on the ventral spots of the frogs.”
		“Frogs call the water with their songs.”
	*Rhinella* spp*.*	“For erysipelas, a living toad is opened and placed on the wound, then the toad is put in the sunlight to dry along with the disease.”
		“Big toads use poison to kill, two dogs have died because of it.”
Reptiles	Snakes (general)	“You can use the venom as an antibiotic.”
		“People use the body fat as a treatment of vascular diseases and the fading of scars.”
		“Snakes cause skin diseases such as sores, rashes, allergies, inflammations, and even skin necrosis.”
		“The snake is rubbed into the varicose vein and let go to take the disease away.”
		“When you kill a snake, you have to hang up the parts away from each other or they join again.”
	*Boa constrictor* Linnaeus, 1758	“The skin is used to make belts.”
		“You can make an ointment and use it for fading scars.”
	*Crotalus durissus* Linnaeus, 1758	“The ground skin, tail, blood and venom are encapsulated and used to cure cancer and influenza.”
		“The rattle serves as an amulet, but if you remove the skin of the snake it is unlucky.”
		“Using the venom, you can make antibiotics and samples to cure diseases.”
		“The rattle is a good luck charm.”
	*Hemidactylus* sp.	“It transmits leprosy and causes burns and skin irritation.”

As for reptiles, 29 of the respondents did not associate this group with their cultural or magical-religious beliefs, whereas three persons said they did not know. Twenty-nine of the respondents described beliefs associated with luck and use in traditional medicine. Fifty-four people of the population surveyed perceive reptiles as dangerous animals and 17 people associate them with the transmission of diseases such as leprosy, burns, and other skin problems.

### Interaction

**Fig. 2:**
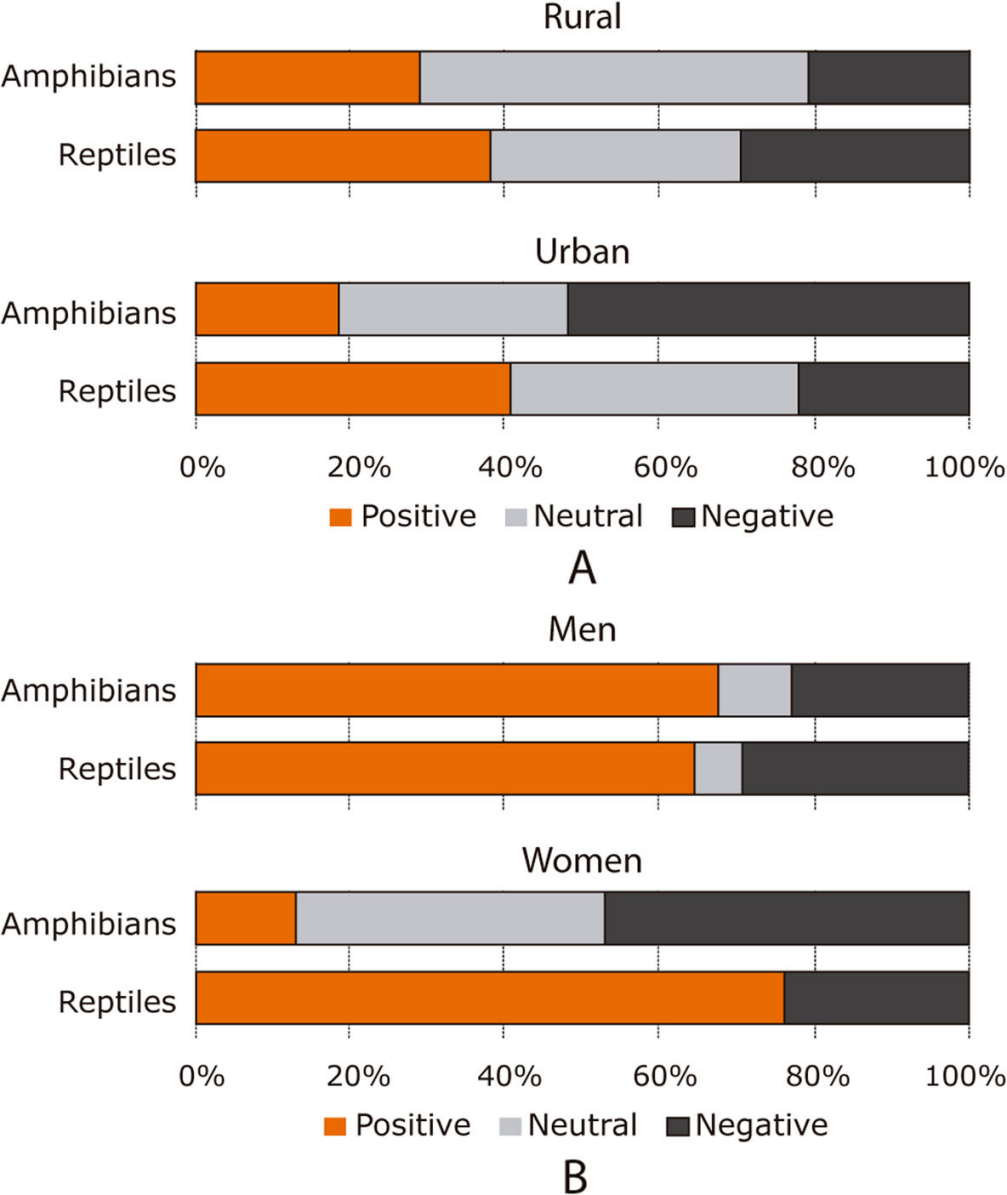
Response tendencies regarding interaction with amphibians and reptiles. It shows the positive, neutral, and negative reaction rates in a possible encounter with amphibians and reptiles regarding the birthplace (**a**) and gender (**b**)

The tendency chart suggests a possible correlation between the demographic variables of birthplace and gender, within the interaction with amphibians. Compared to people living in the rural area, people living in the urban area are more likely to have a hostile reaction in a possible encounter with an amphibian. Also, women tend to have a more negative behavior towards amphibians than to reptiles (Fig. 2).

In contrast, there is only one possible incidence for the gender variable in reptile interaction, where men are more likely to react hostile to an encounter with reptiles than women. In addition, there were no neutral behaviors by women in this variable, being the majority positive reactions (23 of the respondents) and the remaining ones negative (seven women). There was no apparent relationship between age and the interaction with amphibians and reptiles.

For most people surveyed (34 people) the abundance of herpetofauna in the RFPCQ has decreased in recent years, especially amphibians. Twelve people of the surveyed population expressed that abundance has increased, whereas ten persons state that it has remained the same. Finally, five persons said they did not perceive a change. Respondents described agricultural activities, chemical fumigation, deforestation, and water pollution as possible current impacts on the amphibian and reptile community in the region. All respondents recognized the importance of generating conservation programs and sustainable usage of the habitat to protect the herpetofauna in the region.

## Discussion

### Academic knowledge

The surveyed population recognizes the existence of amphibians and reptiles in the region, although little is known about general aspects of their biology and the ecological role of this group, for example, the ability of reptiles to prevent insects pests, such as mosquitoes, or control of populations of other animals such rodents or even snakes [44, 45]. However, the inhabitants properly recognize the potential habitats of these animals, which could help identify priority conservation areas [10, 41, 46, 47].

The local community recognizes the existence of venomous snakes in the region, although it tends to ignore the existence of non-venomous species. This may be due to the association of the body morphology of these animals with risk factors, generally associating serpentiform animals as dangerous; given the evolutionary conditioning of human species [48], which has also been recorded in other primates [49].

Although the population surveyed is aware of the possibility of an ofidic accident, there is a general lack of knowledge about the protocols to follow in case of snake bites, as the use of anti-ofidic serum is uncommon. This lack of knowledge amplifies the human-snake conflict since the most widespread action to avoid ophidian accidents is killing snakes (even non-venomous), making this one of the main threats to the ophidians’ population in Colombia [50].

### Use and cultural beliefs

Nearly half of the people surveyed associated reptiles (exclusively snakes) as a part of ancestral culture and traditional medicine, whereas for amphibians this association was lower. We propose two reasons as an explanation: (1) As in other regions of Latin America, given the traditional representation of power and the strong ligation of snakes with magical-religious beliefs, the development of a worldview that linked snakes to luck and uses in traditional medicine is more common, based on the cure through the power and the spirit that snakes represent even when this group of animals is considered as dangerous for the people in general [2, 45]. (2) Since amphibians are mainly nocturnal, a meeting with people in their daily activities is less common. This has resulted in less strong interaction and a minor influence on the worldview of the inhabitants of the region. Although Panche indigenous communities made drawings of amphibians and associated them with water [20], it seems that these traditions were not maintained over time by the current inhabitants.

A third of the population surveyed perceives amphibians as dangerous and disease-causing animals. The main belief was that skin wounds are caused by urine or secretions of the parotid glands of the toads (*Rhinella* spp.). Although information on the effect of bufotoxins on human health is scarce [51, 52], there are extensive records of intoxication in dogs [53–56] and wild vertebrates [57–59]. This is a problem especially widespread in countries in which this species has been introduced, sometimes causing the death of the animals involved [54, 56]. However, it is more common to find that toads are avoided due to their physical appearance, which, added to the lack of knowledge about their biology, results in the association of these animals with areas of debris and dirt.

On the contrary, most of the population surveyed perceive reptiles as dangerous animals (54 people), based mainly on the fear of an ophidian accident. Although 47.5% of the reported cases of poisoning in Colombia correspond to ophidian accidents [60], between 2014 and 2016 only 185 cases of snakebite were reported in the department of Cundinamarca (61 cases on average per year [61];), it is, therefore, one of the departments with fewer reported cases in Colombia (< 2%). This can indicate that the response of fear towards snakes is more linked to the cultural imprint rather than to the interaction and previous experiences of the inhabitants with snakes in the area.

Despite this, there are several uses of amphibians and reptiles in traditional medicine, such as the use of dissected toads for the cure of varicose veins, shingles (herpetic skin lesion), erysipelas, and other dermal bacterial infections, as well as the use of snakes for the cure of some diseases such as cancer and heart ailments (Table 2). Previous studies have also reported these uses in other Latin American countries [62–64] and other regions of the world [65, 66]. Besides, in recent decades there has been increasing evidence on the possibility of pharmacological synthesis based on chemical components obtained from secretions, tissue, and venom extracted from amphibians and reptiles [67–70], which shows the correspondence between the use of species in the traditional culture, as well as in current and future applications in medicine.

### Interaction

The response of the rural population towards the interaction with amphibians is dominated by neutral and positive behaviors, which contrasts with the response to the same interaction in the urban population (Fig. 2).

For the rural population, amphibians are a fundamental part of the nocturnal sound ecosystem, which could have a positive impact on the perception of these animals. As the occurrence of amphibians in the urban area is less frequent, people likely develop a negative perception due to the lack of contact. On the contrary, the trend response to interaction with reptiles between the rural and urban population is similar, which could indicate that there is an imprint of information on reptiles that remains more stable between the community and birthplaces.

Despite people’s cultural/natural fear towards snakes [48, 71], in most cases, the positive behavior in the interaction with reptiles was higher than in the interaction with amphibians. However, this may be due to the difference in the classification of positive and neutral reactions, where the behavior of standing still or moving away is considered neutral for the interaction with amphibians, whereas it is positive for the interaction with reptiles. The above is based on the idea that possible positive behaviors towards reptiles (specifically snakes) do not consider transporting the animal to a safe place, due to its potential danger [71–73].

Men described mostly positive behaviors in response to interaction with amphibians and reptiles, tending to have few neutral behaviors in both groups. This indicates that, although snakes are perceived as dangerous animals, this usually does not lead to defensive behavior that implies the threat of species in the study region, a phenomenon previously reported in other parts of Latin America [2, 45, 71, 73] where the conservation status of different species is put at risk by the effect of the controlling hunt and the use of this group of animals with magical-religious purposes. In contrast, women in the surveyed population tend to have more negative behaviors in the interaction with amphibians compared to reptiles. This may be related to the possible danger of snakes, since taking a negative reaction and attacking the animal implies a health risk, which does not occur with amphibians and could make them more susceptible to be attacked.

Although the data provided by this study represent a preliminary approach to the understanding of current human-herpetofauna interactions in the RFPCQ, the implementation of a more direct methodological design is necessary, to test the reactions of people in a controlled environment. These studies are vital as a basis for planning conservation strategies in the region since the interaction of humans with nature directly influences the survival dynamics of current animal populations [64, 74, 75].

### Final considerations

When local communities are empowered with basic knowledge about the species that occur in their region and become active agents of participation for their conservation, management, and preservation, plans are effective [2, 46, 47, 76]. The local population recognizes the importance of preserving and conserving nature, although they are unaware of the fundamental reasons why this type of action should take place. Thus, processes such as deforestation, degradation, and habitat loss caused mainly by human activities such as improper use of land, overexploitation of natural resources, and the exclusivity of land use for agriculture and cattle ranching need to be reassessed [75, 77].

In this way, ethnozoological studies become relevant by serving as a bridge that unites the different communities in favor of the conservation of species, making them active agents in all phases of these processes [2]. Studies of this type allow us to give an account not only of which species are used by the communities but also of how they are used, taking into account the impact of this human-animal relationship on the implementation of conservation plans for different species, many of which are used intensively and/or in danger of extinction [5, 64, 74, 78]. Furthermore, these studies offer local ecological information on important species, strengthening the discussion about their conservation [79, 80] without limiting the access of local communities to fauna and allowing the prioritization of species in such conservation plans, as proposed by Martínez (2013) [81] in the Argentine Chaco region.

Similarly, it is possible to develop conservation plans involving a greater number of actors through ecotourism, which serves as a source of income for local people and as a means of conserving entire ecosystems, using the experience of the communities in the execution of these plans. This practice is known as ethnotourism, which has been gaining strength as a conservation strategy [82, 83].

Due to the current way in which science is communicated, with a vast majority of scientific papers not being very informative for local communities, it is common to find only a limited scope of knowledge in local communities, not allowing their participation in decision-making [2, 84]. Although biology is a discipline that uses rather technical terms, efforts to ensure assertive communication must be prioritized to allow the reduction of sectorization and to make information available to the people most directly related to the natural environment [45, 85].

## Conclusion

Although there was a general lack of knowledge on the part of the inhabitants of the RFPCQ about the biological and ecological aspects of herpetofauna, the population recognized the basic information about the habitats of these animals within the area of the reserve, which could help identify possible priority conservation areas.

Most of the population does not associate the herpetofauna with cultural or magical-religious traditions. However, there is a wide variety of uses of amphibians and reptiles in traditional medicine, which are consistent with the information obtained in other regions of Latin America and the world.

In general, the perception of amphibians and reptiles varied according to the origin and gender of the people surveyed, tending to have a more positive perception about reptiles that of amphibians in most cases.

Greater efforts should be made in the transmission and dissemination of knowledge about the ecological functions of herpetofauna, which will contribute to the success of future conservation plans in the area with the local community’s participation.

## Supplementary information

**Additional file 1.** Survey format

**Additional file 2.** Surveys data

## Data Availability

A complete survey model can be found as Additional file 1. Survey format conducted on the inhabitants in the study area.docx. Data collected from surveys are available in Additional file 2. Surveys data.xlsx.

## Abbreviations

RFPCQ: “Reserva Forestal Protectora Cerro Quininí” forest reserve
Sp (p): Species
