# Supplementary material for: Knowledge and interactions of the local community with the herpetofauna in the forest reserve of Quininí (Tibacuy-Cundinamarca, Colombia)
Source: J Ethnobiol Ethnomed. 2020 Apr 15;16:17. doi: 10.1186/s13002-020-00370-8 (PMC7161309; doi:10.1186/s13002-020-00370-8)
Supplement: Supplementary file 1 — Additional file 1. Survey format [file 13002_2020_370_MOESM1_ESM.docx]

# Additional Files

**Additional file 1.** Survey format conducted on the inhabitants in the study area.

| **Question** | **Answer** |
| --- | --- |
| **Demographics** | |
| Name |  |
| Gender |  |
| Age |  |
| Education Level |  |
| Occupation |  |
| 1. **Academic knowledge** | |
| Have you ever heard of poison frogs or toads? | Yes/No |
| Do you think toads or frogs are important to preserve nature? | Scale from 1 to 5, with 1 being the most negative and 5 being the most positive. |
| Do you know the role that toads and frogs play in nature? | Yes/No  Which one? ____________________________________________________________________________________________________________ |
| Do you know how toads or frogs breed? | Yes/No  How?___________________________________________________________________________________________________________________ |
| Which of the following groups would you place a toad or frog in? | (A) Amphibians, (B) Reptiles, (C) Does not know |
| Which of the following sites do you believe or have seen that toads or frogs usually remain? | (A) Very tall trees, (B) Dry soils, (C) Wet soils, (D) Crops, (E) Dumpsters, (F) Plants with water reserves. |
| Do you think water is important for the life of a toad or frog? | Yes/No  Why?___________________________________________________________________________________________________________________________________ |
| Have you ever heard of poisonous snakes in your region? | Yes/No |
| Do you know the term anti-ophidic serum?  Do you know how to use it, or have you used it? | Yes/No |
| Do you think snakes or lizards are important for the preservation of nature? | Scale from 1 to 5, with 1 being the most negative and 5 being the most positive. |
| Do you know the role reptiles (lizards and snakes) play in nature? | Yes/No  Which one? ____________________________________________________________________________________________________________________ |
| Do you know the role reptiles (lizards and snakes) play in your region? | Yes/No  Which one? ____________________________________________________________________________________________________________________ |
| Which of the following groups would you place a snake or lizard in? | (A) Amphibians, (B) Reptiles, (C) Does not know |
| Which of the following sites do you think a snake could be found on? | (A) Very tall trees, (B) Dry soils, (C) Wet soils, (D) Crops, (E) Dumpsters, (F) Plants with water reserves. |
| 1. **Cultural use and beliefs** | |
| Have you ever heard of any illnesses a person has contracted from touching a toad or frog? | Yes/No  Which one? ____________________________________________________________________________________________________________________ |
| Do you know about any cultural tradition associated with a toad or frog? | Yes/No  Which one? ____________________________________________________________________________________________________________________ |
| Have you heard of or know of a traditional or home remedy in which a toad or frog is used? | Yes/No  Which one? ____________________________________________________________________________________________________________________ |
| Have you ever heard of any illnesses a person has contracted from touching a snake or lizard? | Yes/No  Which one? ____________________________________________________________________________________________________________________ |
| Do you know about any cultural tradition, myth or belief associated with a snake or lizard? | Yes/No  Which one? ____________________________________________________________________________________________________________________ |
| Have you heard of or know of a traditional or home remedy in which a snake or lizard is used? | Yes/No  Which one? ____________________________________________________________________________________________________________________ |
| 1. **Interaction and perception** | |
| If you found a toad or a frog in your home or in an area adjacent to it, what would you do? | (A) I would try to kill it, (B) I would try to scare it, (C) I would simply come forward to observe it, (D) I would catch it and look for a body of water nearby to leave it there, (E) I would do nothing, (F) Other, Which? _____________ |
| Do you know of any cases in which a person has been bitten by a snake? | Yes/No |
| Do you think it is important to preserve the sites that inhabit snakes? | Yes/No  Why? ____________ |
| In the case of meeting a snake, what would you do? | (A) I would walk away or escape, (B) I would try to kill it, (C) I would try to scare it away, (D) I would just come over to observe it, (E) I would catch it and look for a safe place to leave it there, (F) Nothing, (G) Other What?_________ |
| Over time amphibians and reptiles have: | (--) Decreased a lot, (-) decreased, (-) remained equal, (+) increased, (++) greatly increased, (NS) Does not know. |
| Do you think habitat preservation, use, and care programs should be done to protect amphibians and reptiles in your region? | Scale from 1 to 5, with 1 being the most negative and 5 being the most positive. |
| Do you think that performing anthropic (man-made) activities such as crops, tree felling or water contamination, among others, may be affecting the populations of amphibians and reptiles associated with your region? | Scale from 1 to 5, with 1 being the most negative and 5 being the most positive. |
| Do you think toads or frogs can be dangerous to you or a family member? | Scale from 1 to 5, with 1 being the most negative and 5 being the most positive. |
| Do you think snakes may be dangerous to you or any member of your family? | Scale from 1 to 5, with 1 being the most negative and 5 being the most positive. |
| Do you know the procedures necessary for the care of a person who has been bitten by a snake? | Yes/No  Which one? _____________ |
